# Breaking barriers in *Candida* spp. detection with Electronic Noses and artificial intelligence

**DOI:** 10.1038/s41598-023-50332-9

**Published:** 2024-01-10

**Authors:** Michael L. Bastos, Clayton A. Benevides, Cleber Zanchettin, Frederico D. Menezes, Cícero P. Inácio, Reginaldo G. de Lima Neto, José Gilson A. T. Filho, Rejane P. Neves, Leandro M. Almeida

**Affiliations:** 1 Centro de Informática, Universidade Federal de Pernambuco, Recife, PE Brazil; 2 Comissão Nacional de Energia Nuclear, Centro Regional de Ciências Nucleares do Nordeste, Recife, PE Brazil; 3 Departamento de Mecânica, Instituto Federal de Pernambuco, Recife, PE Brazil; 4 Centro de Ciências Médicas, Universidade Federal de Pernambuco, Recife, PE Brazil; 5 Centro de Ciências Sociais e Aplicadas, Universidade Federal de Pernambuco, Recife, PE Brazil

**Keywords:** Computational biology and bioinformatics, Infectious diseases, Scientific data

## Abstract

The timely and accurate diagnosis of candidemia, a severe bloodstream infection caused by *Candida* spp., remains challenging in clinical practice. Blood culture, the current gold standard technique, suffers from lengthy turnaround times and limited sensitivity. To address these limitations, we propose a novel approach utilizing an Electronic Nose (E-nose) combined with Time Series-based classification techniques to analyze and identify *Candida* spp. rapidly, using culture species of C. *albicans*, C.*kodamaea ohmeri*, C. *glabrara*, C. *haemulonii*, C. *parapsilosis* and C. *krusei* as control samples. This innovative method not only enhances diagnostic accuracy and reduces decision time for healthcare professionals in selecting appropriate treatments but also offers the potential for expanded usage and cost reduction due to the E-nose’s low production costs. Our proof-of-concept experimental results, carried out with culture samples, demonstrate promising outcomes, with the Inception Time classifier achieving an impressive average accuracy of 97.46% during the test phase. This paper presents a groundbreaking advancement in the field, empowering medical practitioners with an efficient and reliable tool for early and precise identification of candidemia, ultimately leading to improved patient outcomes.

## Introduction

Infections caused by fungi are a significant issue in the scenario of Intensive Care Units (ICUs), increasing morbidity and the number of deaths in patients who are in a critical state of health^1,2^. The main reason for the occurrence of this type of infection, also described as invasive fungal infections (IFI), is candidiasis, with *Candida albicans* as the primary causative agent, followed by *Candida parapsilosis*, *Candida glabrata*, *Candida krusei* and *Candida tropicalis*^3^. According to reports by^4^, approximately 15 species of *Candida* can cause human diseases, and the most common, presented in more than 90% of cases. Furthermore, there have been notable changes in this field, with the emergence of species considered rare or uncommon, such as occurrences with *C. pelliculosa*, *C. haemulonii*, *C. guilliermondii*, *C. lusitaniae*, *C. famata *and *C. auris*^4,5^.

Data reported by^5^ show that, despite considerable advances in antifungal therapy in recent years, mortality related to Invasive fungal infections (IFIs) in ICUs has been 40 to 60%. One of the factors contributing to this mortality rate is the challenge in recognizing and diagnosing IFIs in the early stages of treatment^5,6^. According to^6^, only half of the tested patients were reported to be infected by *Candida* spp. Considering that the result may take 2 to 7 days to be confirmed (in the case of culture-based methods), and given the severity of this condition, a delay of more than 12 hours can increase the risk of mortality.

At present, blood culture is the standard method in the laboratory diagnosis of candidemia, enabling the isolation of the causative agent for identification^7^. Alternative techniques that do not rely on cultures are also used, including polymerase chain reaction (PCR), detection of mannan and beta-D-1,3-glucan antigens (BDG), and enzyme-linked immunosorbent assay (ELISA). It is important to note that some of these approaches involve careful sample preparation, have long response times, entail significant costs, and require professionals with specific expertise^8,9^.

In addition, we can also mention T2Candida, which combines targeted PCR with T2 magnetic resonance and Matrix-assisted laser desorption/ionization-time of flight (MALDI-TOF) mass spectrometry (MS). T2Candida allows for early detection of candidemia in patients undergoing antifungal therapy; however, it is not suitable for low-prevalence environments, is costly, and covers only five of the main species^10^. As for MALDI-TOF MS, it is highly successful in identifying clinical samples, but it can be a time-consuming process and heavily relies on the expertise of clinical mycologists handling the samples^8,12^. Furthermore, according to^13^, combining the MALDI-TOF MS technique with other methods is often advisable to achieve more accurate and satisfactory results. However, this approach also involves the use of equipment that can be costly and may not always be readily available in various microbiology laboratories, particularly in developing countries^14^. However, alternative methods are based on detecting Volatile Organic Compounds (VOCs) to identify these fungal agents. These methods include Gas Chromatography-Mass Spectrometry (GC-MS), Solid Phase Microextraction (SPME), Simultaneous distillation extraction (SDE), and Selected Ion Flow Tube Mass Spectrometry (SIFT-MS)^15^.

Another method that has received some attention and shows potential for development is the Electronic Nose, often called the “E-Nose.” This technology combines a variety of gas sensors and uses artificial intelligence to identify patterns of Volatile Organic Compounds (VOCs) and categorize the unique “smell fingerprints” associated with these compounds. This tool is generally built with metal oxide conducting chemical sensors (MOS), which are responsible for identifying the volatile organic compounds released by the odor-emitting components. Its functioning is based on the olfactory function of mammals and has been studied since the 1980s^16^. Like a real nose, the E-nose aims to identify patterns from the VOCs identified by the sensors, whose reading values are analyzed and classified by an artificial intelligence (AI) model. This device typically comprises three main parts: sensors, a signal processing unit, and a pattern recognition system^17^.

The Electronic Nose is already being applied in various domains, from food safety to agricultural applications and disease diagnosis, as^18^ mentioned. For a more comprehensive view of these applications, one can delve into studies conducted by^19,20^, and^21^, which focus on the identification of microorganisms, including fungi and bacteria. Furthermore, research carried out by^22,23^, and^24^ further extends the exploration of Electronic Nose applications in the food industry. It’s also worth highlighting the study by^25^, in which a portable Electronic Nose device is employed to diagnose gynecological conditions in a clinical setting rapidly.

In the context of medical diagnosis, Electronic Noses have experienced remarkable advances in recent years, particularly in hardware development and algorithm evolution^18,26^. Medical diagnosis stands out among the fields most benefited by the progress of this technology, as previously mentioned^18^. However, some limitations still require refinement, such as the stability, standardization, and reliability of certain sensors^27,28^. In this regard, efforts are being devoted to enhancing the sensitivity, selectivity, and stability of these devices, with significant progress when these mechanisms are integrated with artificial intelligence and Machine Learning techniques^18,29^.

Given the above, it is understood that there is a significant issue regarding the rapid identification of fungi in hospitalized patients and those with a clinical condition that requires extra care^30,5^. Considering that this identification process can be improved, this project proposes using an Electronic Nose to recognize patterns related to fungi of the *Candida* spp. species^31^ utilizing control samples collected by ATCC company. This method can be combined with a set of machine learning techniques, enabling quicker and more efficient identification^32^, streamlining the decision-making process of health professionals, and, consequently, improving the survival chances of these patients. It is essential to mention that in this initial proof-of-concept study, we are using only culture samples, aiming at the creation and validation of a rapid and efficient protocol that can be replicated in the future for samples of other materials, such as whole blood. To better understand this, the following sections will address the Materials and methods used for the construction of the study, the Results and discussions on its development, and, finally, the Conclusions of the findings of this investigation.

## Results

Through the implemented models, a series of experiments were conducted and cataloged using the metrics Accuracy, F1-score, Recall (Sensitivity), Specificity, Precision, and Standard deviation, aiming to identify patterns in the VOCs released by the analyzed *Candida* species. The variety of models covered the different characteristics that the data may have, highlighting the models that best fit the data standard and discarding those with less potential. Initially, all models were applied with the parameters defined by the documentation or in their respective repositories. The possibility of including a parameter validation step for the models was considered. However, given the satisfactory performance of most models and considering the computational cost and time that this step would require, it was deprioritized for the time being.

Regarding the methods used, the primary rationale for using time series models is the temporal nature of the signal reading, with data from each round of the aspiration process being added to the database. The majority of the models used were sourced from the Sktime library. However, due to its uniqueness, Inception Time was the only one implemented independently of the library, as there is currently no tool that simplifies access to its functions and properties. The model code provided by the authors on GitHub had to be modified to accommodate the metrics and dataset of this study.

As a result of the training stage, most of the models achieved 100% accuracy. This is justified due to the reduction of instances that the pre-processing step brought, using the cycles as training elements. Thus, models learn data patterns better as they have less to memorize. In this regard, to prevent overfitting, in addition to adding more data cycles for model training, grid search steps or optimization algorithms can be employed to find better parameters^33^. Another commonly used strategy is the application of more robust models, as was the case with InceptionTime^34^, achieving greater consistency at all stages of the process.

In addition to the average value referring to the metrics in the training process, the values referring to the averages of the validation and testing stages of the models were also recorded. There was a moderate decrease in the performance of the models between the training phase and the validation and test phases, amidst 7 and 4%. This is because, during training, the models identify *Candida* patterns precisely due to the distinctive nature between each species and the new data division. In the other phases, as they are new data, and the model has never seen them, it is normal and expected that it ends up making more errors, which in no way interferes with its final evaluation. Table  1 demonstrates the data referring to the testing steps of each of the models.

**Table 1:** Result of the model testing stage—test values for the metrics Accuracy, F1-score, Recall (sensitivity), Precision, Specificity, and Test time measured for each model after the training and validation phase.

Classifiers	Accuracy	F1-score	Recall (Sensitivity)	Precision	Specificity	Test time (s)
Inception Time	**0,97468**	**0,97605**	**0,97817**	**0,97540**	**0,99513**	1,21489
Random Interval Spectral Ensemble (RISE)	0,65000	0,55758	0,57007	0,56251	0,94223	4,58585
Time Series Forest Classifier	0,67500	0,61261	0,58960	0,61261	0,91312	1,18719
ROCKET Classifier	0,78750	0,78105	0,85764	0,79171	0,93804	4,91326
Shapelet Transform Classifier	0,63750	0,58207	0,60258	0,59832	0,93028	0,61583
K-Neighbors Time Series Classifier	0,75000	0,73245	0,72192	0,81357	0,95635	52,86100
HIVE COTE 1	0,52500	0,40245	0,42669	0,39475	0,94009	11,93961
HIVE COTE 2	0,66250	0,58503	0,60360	0,62833	0,94342	2,57620
BOSS Ensemble	0,63750	0,50525	0,53555	0,49929	0,90165	**0,48432**

Significant values are in bold.

As observed in the result set, the most notable model was Inception Time^34^, executed with the standard set of parameters, followed by ROCKET Classifier^35^, Time Series Forest Classifier^36^ and Random Interval Spectral Ensemble (RISE)^37^, respectively. All metrics calculated in Inception Time were near 100%, demonstrating high consistency between the results.

In addition to collecting the metrics, statistical tests were conducted to verify the difference between the results of the different models. Specifically, a normality test was performed with the accuracy results obtained in the 10 repetitions for each model of the validation stage. This was followed by a significance test and a post-hoc test to compare the selected algorithms pairwise.

It can be interpreted that only the Inception Time model does not follow a normal data distribution. It would already suggest using a non-parametric test to evaluate the results. However, to obtain increased sensitivity of the analyses, a numerical test of statistical normality was also applied, where the most suitable test for the problem in question was the Shapiro-Wilk test. According to^38^, this method is more suitable for small sample sets smaller than 50, although it can also be used for larger sets. In contrast, methods such as Kolmogorov-Smirnov are ideal for samples larger than or equal to 50. Both tests use as a null hypothesis the statement that the data are all derived from a normal distribution set, accepting this hypothesis when p>0.05, confirming the data as normally distributed.

As a result of applying the normality test, the HIVE COTE1, Shaplet Transform Classifier, and TimeSeries Forest Classifier classifiers did not present a normal distribution according to the Shapiro-Wilk test, with p-values equal to 0.01227, 0.03521, and 0.00021, respectively. All these values are less than 0.05.

Indeed, with this result, we confirm the need to apply a non-parametric test, given that only some groups follow a normal distribution. As per^39^, the most appropriate non-parametric test for this case is the Kruskal-Wallis test, considering the number of examples in the groups is small and equal. For the execution of the test, the following hypotheses were considered:

- H0: All models have relatively equal means in terms of classification accuracy;
- H1: At least one of the models differs from the others in terms of mean classification accuracy.

Where H0 is the null hypothesis, which assumes that all models have equal performance, H1 is the alternative hypothesis, which is the difference in performance of at least one of the models about the others. For this test, a p-value less than 0.05 indicates the rejection of the null hypothesis, suggesting the existence of a significant difference between the evaluated samples. Thus, applying Kruskal-Wallis to the set of results acquired, a p-value of 2.49E-02 was obtained, which is less than 0.05. This demonstrates that with 95% confidence, there is evidence to reject H0 and accept the hypothesis that at least one of the models differs from the others in mean validation accuracy.

**Figure 1:**
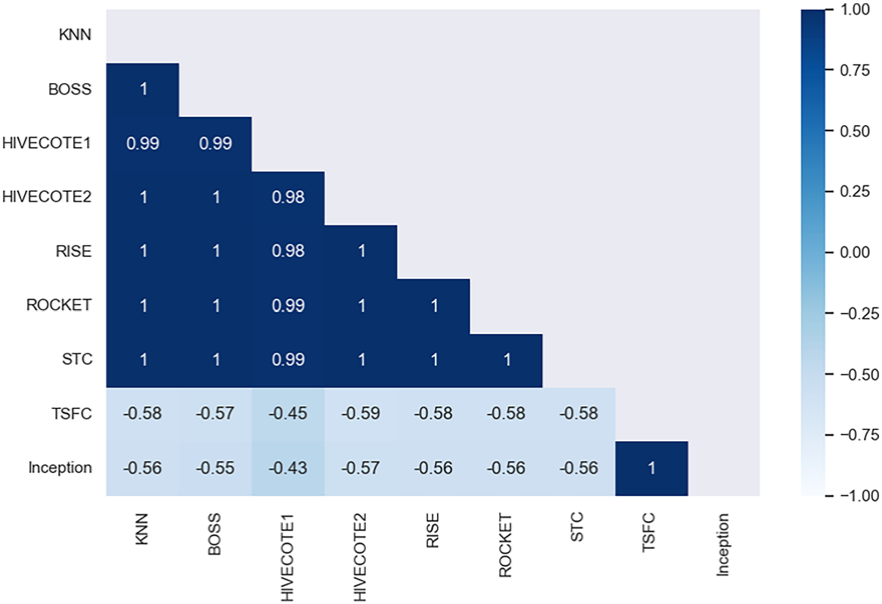
Correlation graph of the results of applying the Nemenyi post-hoc test on the set of results for each model. In this type of graph, when it is farther from 1, the elements are more divergent; that is, they are statistically different.

Given this model difference, the next step was applying a post-hoc test to identify which models are statistically different. The non-parametric test only indicates the existence of this difference, not the relationship between the sets. For this step, the Nemenyi test was used, which, according to^40^, is one of the most commonly used post-hoc tests after applying Kruskal-Wallis. As briefly explained, this method performs a pairwise investigation of each analyzed set, returning the p-values for each relationship between the evaluated groups. The values vary between -1 and 1, with p<0.05 indicating a significant statistical difference between the samples according to the test and values closer to 1 demonstrating similarity. Figure 1 depicts a correlation matrix that crosses the results obtained by the Nemenyi method.

As observed, there is a high similarity between most models with a lower accuracy average, not showing a significant statistical discrepancy between them. However, it can be stated that there is no significant difference between the Inception Time^34^ and Time Series Forest Classifier^36^ models, both of which have p-values much less than 0.05. Each one has a notable difference between the models with more distinct accuracy. However, there is a high similarity between some with closer accuracy (which was expected), dividing the models into different groups of relevance. This way, it is possible to identify the difference between the models, with Inception Time and TSFC not showing a significant difference. Although there is no significant statistical difference, Inception Time stands out with average values for Accuracy, Precision, Recall (sensitivity), Specificity, and F1 above 95% in all analysis sets. Additionally, it boasts an execution time of just over 1 second, making it the most promising choice for the final classification model of volatile compounds emitted by *Candida* species. The entire process of identifying microorganisms, encompassing sample reading and model classification, is completed in approximately 15 minutes.

## Discussion

With the results of this study, it is possible to see the effectiveness of using Electronic Noses in the face of such complex problems, including identifying fungi through VOCs emitted by species in culture. In contrast to other solutions, using this technology, in addition to making the process of helping identify fungi cheaper, can speed it up, achieving a satisfactory result within a few hours. Traditional methods use expensive, large machines (challenging to transport), which require a longer time to indicate an accurate result. With the E-nose built with low-cost parts in a compact suitcase, it will be possible to transport it more easily and quickly. The identification speed is up to the AI models being trained because the more accurately they use data with less culture time, the faster their classification returns.

From the first stages of the study, in the visual analysis of the data, it is possible to identify a distinct separation between some species (highlighted in the PCA of the Fig. 4a). This helps to understand which *Candida* species can be better identified by the models and demonstrate a linear separation between some. For example, it’s possible to observe in the left part of the projection a cluster of five species (C. *albicans*, C. *glabrata*, C. *haemulonii*, C. *kodamaea ohmeri*, and C. *krusei*), which could be separated by some lines, as well as in the lower right corner, where C. *parapsilosis* and C. *krusei* are located, and in the upper right corner, where C. *albicans* and C. *glabrata* can be found. It’s worth noting that some other species within these groups might account for some of the errors recorded by the models during the learning process.

Another critical point is the choice of Time Series for training and data classification. This decision was taken given the temporal characteristic of the data, both for the time of culture of the fungi and for the reading of the volatile emitted by them and captured by the Electronic Nose, based on the process in evidence in Fig. 3c.

All this flow culminated in obtaining outstanding results for the validation and classification phase of the samples, where most of the models achieved an assertiveness above 90%, with emphasis on the Inception Time, with an average of 97.70%, 95.87%, and 97.46% of accuracy in the training, validation, and testing phases, respectively, with very similar values for the other metrics. In the training step, most models reached 100% in all metrics. However, this can be seen as a bias in the data, harming the test step. All this difference was confirmed by the analysis of statistical significance, where through the Shapiro-Wilk normality tests, the Kruskal-Wallis non-parametric test, and the Nemynyi post-hoc test, the difference between the algorithms used was identified.

Although there are still no comparative studies between the E-nose and artificial intelligence in relation to more traditional yeast identification techniques, we can observe a great similarity between the efficiency of the method presented in this work and methods such as MALDI-TOF MS, CHROMagar and Corn meal tween-80 agar, as demonstrated in study^41^. The authors’ approach indicates that, even though these techniques are not considered gold standard for yeast identification, they can lead to very promising results for some species, with a performance very similar to that of our study (indicated in Table 1), when compared to the percentage of correct answers. This highlights the importance of using new methods that can fill the gaps left by more traditional methods.

Thus, as seen in the study, it is possible to perceive how powerful Electronic Nose, combined with new Time Series techniques, can yield satisfactory and promising results. Because it is a portable tool with a moderate construction cost compared to current mechanisms - it can reach a wide range of environments in places with fewer resources and difficult to access. These facilitators should broaden the identification process’s scope of use, benefiting many people. For the next steps, samples of new species of *Candida*, even rarer, such as *C. tropicalis*, *C. auris*, *C. famata*, *C. pelliculosa*, *C. guilliermondii*, *C. lusitaniae*, and other fungal segments should be added to the dataset, seeking to create more generic and accurate models in the identification of this fungus. Additionally, these new samples will allow for a broader development of specificity tests among fungi, aiming to ensure the absence of false positives in our results. Furthermore, new analyses will be conducted with shorter culture times to determine if further reducing the identification time is possible.

Another critical step for the future will be to expose the Electronic Nose to patient blood and *in situ* samples to identify its efficiency in a scenario closer to its final operation. In this sense, the equipment requires an environment free from high levels of odors to prevent the risk of incorrect readings due to external interference. However, it can be used in a clinic if there is assurance of an environment free from other contaminating odors (e.g., alcohol, perfumes, air fresheners, etc.). This condition may be possible by using a room containing an extractor fan. From there, health professionals will also perform a qualitative assessment to obtain feedback related to the results indicated by the tool.

## Methods

This work is an evolution of the project developed by^19^, which introduced research on using Electronic Nose and AI to identify *Candida* spp. In the current project, more robust, automated equipment is used that makes it possible to analyze a greater volume of samples. In addition, it also allows the construction of a database of volatile signature patterns and employs advanced AI methods based on Time Series classification. The entire study was developed based on an iterative process of activities, where their execution led to the construction of the final solution. All the code can be found on GitHub (link: https://github.com/michaellopes16/CandidaTimeSeriesClassification.git). It was developed using the Python language on the Jupyter Notebook (in a Core i7 PC, with 16GB of RAM and the GTX 1060 video card) and Google Colab platforms (in your free version). In the initial phases of the research, the primary purpose was to conduct exploratory studies using literary reviews about the main issues related to the work to understand better the state of the art and the best practices for developing the project. In this sense, the course of this section is divided into four stages: Structure and operation of E-nose, Process of sample identification, Analysis and processing of data, and Process of classification of samples.

All these steps seek to select the most promising algorithm for classifying volatiles. In this sense, after the model has been defined, in-place tests must be carried out to ensure its effectiveness in an operational environment. From this, it will be necessary to perform a descriptive study on the use of the solution, aiming to thoroughly analyze its use and better understand its absolute power of contribution, also inserted in this context, a quali-quantitative approach regarding the evaluations.

**Figure 2:**
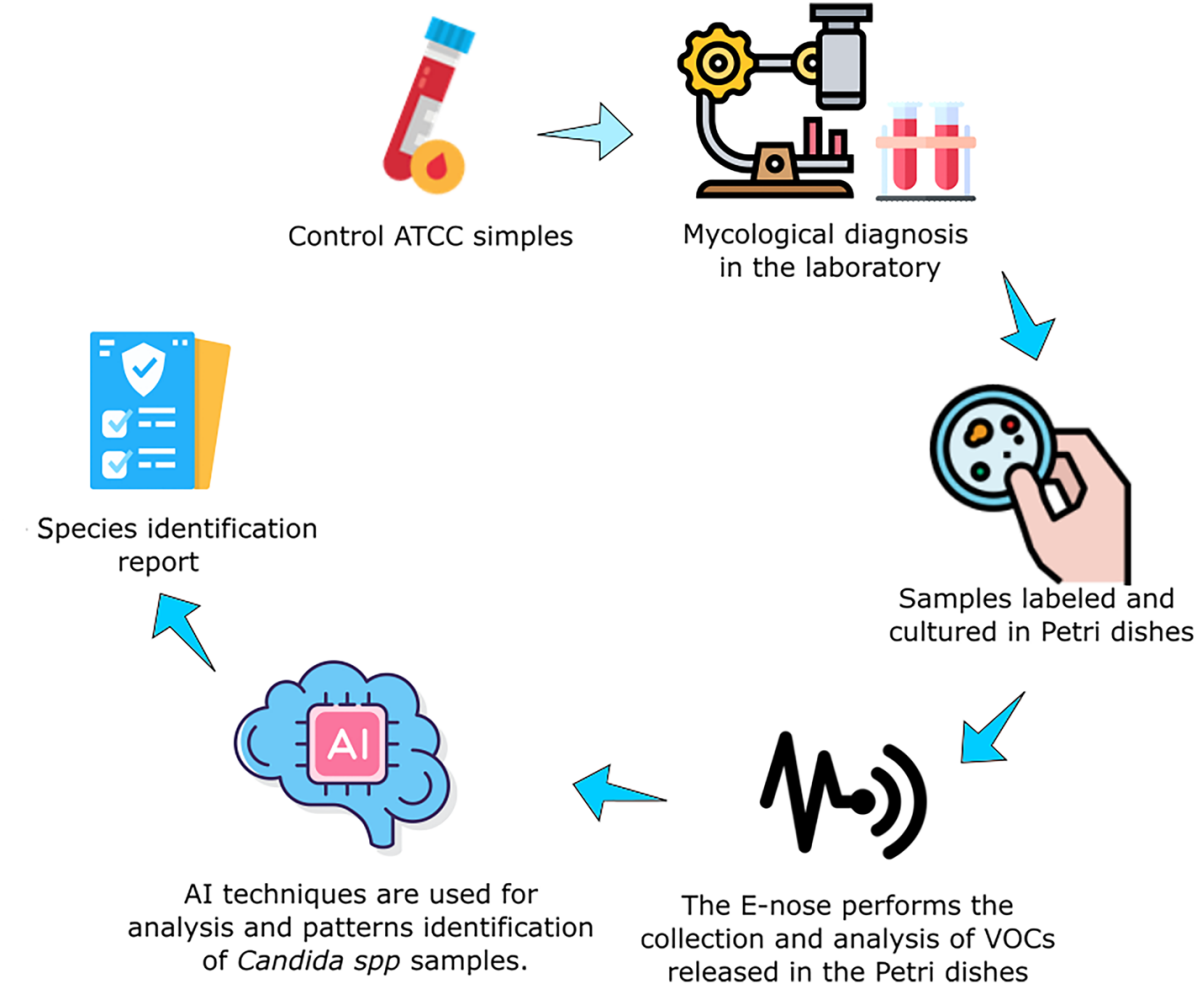
Flow for sample identification and classification. First, control samples derived from the ATCC company are used to analyze and define the mycological diagnosis by the Laboratory of Medical Mycology. With this, the already cultivated species are identified and separated in *Petri* dishes. These cultures are then placed in the E-nose to identify the VOCs. With the collected data, pre-processing routines are executed to use the data already treated by the AI models. At the end, a species identification report is generated.

In this scenario, the project is being developed in partnership with the Mycology department at [Anonimous]. In addition, international alliances are already being prospected, so the collection of samples with different variations can also compose the database under development. The qualitative study should be accomplished through interviews with health professionals to understand the proposal’s feasibility better and identify improvement points. Fig. 2 illustrates part of the process related to the sample identification flow, starting from the Acquisition of control samples to the Species identification report.

### Structure and functioning of E-Nose

In parallel with constructing the theoretical basis and structuring the problem, the first steps for making the solution were carried out. The database was built from control samples created by ATCC (https://www.atcc.org/about-us), an American company offering quality products and services to the scientific and academic community involving biological materials. These samples were utilized by the Laboratory of Medical Mycology/[Anonimous] for the mycological diagnosis. Then, they were labeled and cultivated in Petri dishes for analysis by the Electronic Nose, developed in partnership with the [Anonimous]. The *E-Nose* identifies the “smell fingerprints” released by the fungi through the Volatile Organic Compounds. In this process, the *E-Nose* uses ten different categories of sensors, seven of them from the manufacturer Figaro Engineering Inc. (TGS826 (Ohm), TGS2611 (Ohm), TGS2603 (Ohm), TGS813 (Ohm), TGS822 (Ohm), TGS2602 (Ohm), TGS823 (Ohm)). The other three are the temperature sensors (Co), pressure (kPa), and humidity (%), used to analyze possible interference of these parameters in the behavior of the samples. A summary of the main functions of the sensors used in the device is in Table 2

**Table 2:** Sensors used in the Electronic Nose to identify volatiles emitted by gases generated by the *Candida* species and their functions.

Sensor	Main Function
TGS826	Ammonia detection
TGS2611-E00	Methane detection
TGS2603	Detection of odors and air contaminants (High sensitivity to series of amines and gases with sulfurous odor and high sensitivity to food odors)
TGS813	Detection of combustible gases (High sensitivity to methane, propane, and butane)
TGS822	Detection of Solvent Vapors (High sensitivity to alcohol and organic solvent)
TGS2602	Detection of air contaminants (High sensitivity to gaseous air contaminants)
TGS823	Detection of Vapors from Organic Solvents (High sensitivity to vapors from organic solvents such as ethanol)

**Figure 3:**
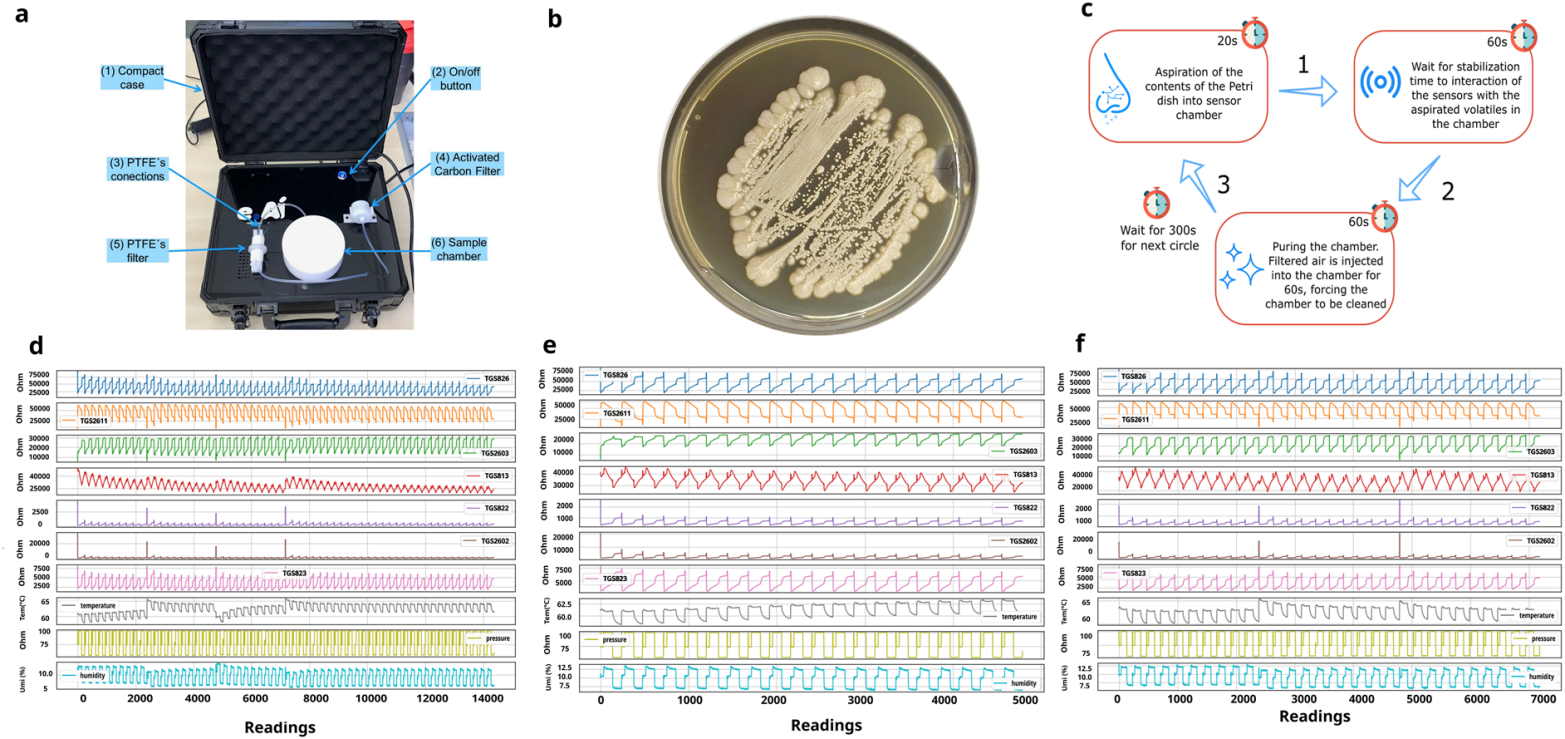
(**a**): Electronic Nose device used in experiments: (1) The Electronic Nose is packaged in a compact box; (2) The on-off switch activates it; (3) All connections are made of PTFE; (4) It has activated carbon filter and (5) PTFE filter; (6) Sample chamber also made of PTFE. (**b**): Example of samples of *Candida albicans* (URM8368) used to create the database. All were cultivated in Petri dishes using Sabouraud Dextrose agar culture medium. (**c**):*E-Nose* collection cycle. (1) Camera suction step (2) Sensor stabilization step (3) Camera cleaning (purge). (**d**): Data from the readings of each sensor over time for the samples of *C. albicans*. (**e**): Data readings from *C. krusei* after one day of culture. (**f**): Data readings from *C. krusei* after two days of culture.

To provide greater flexibility in transporting the device, it was built and adapted inside a compact case, with the appropriate seal and structure to withstand all the elements necessary for the Electronic Nose to work. In this case, in addition to the sensors attached to an air chamber on the inside and the on/off button, there is a pump responsible for the suction/injection of gases or air into the chamber, a control valve, and an air filter with activated carbon and, finally, a simple chamber for inserting the Petri dish and collecting the volatile emitted by the microorganisms’ reactions. All connections between components and chamber surfaces are made with polytetrafluoroethylene (PTFE) due to its non-stick properties and low coefficient of friction, facilitating cleaning and avoiding the permanence of volatiles between the suction and purge cycles. Fig. 3a presents the Electronic Nose Device used in the experiments.

### Sample identification process

As briefly mentioned, the first stage of the sample identification process is accomplished by the Medical Mycology Laboratory/[Anonimous], which manipulates samples. After that, the material is labeled with their respective species, cultivated in Petri dishes containing the culture medium Sabouraud Dextrose Agar (see Fig. 3b with an example of samples of *Candida albicans* (URM8368)) and taken for reading by the Electronic Nose, resulting in the generation of the database. The VOCs of species are aspirated with different culture times to increase the heterogeneity of the data and allow better generalization by models in the future. This aspiration at other times also aims to identify whether it is possible to obtain accurate results faster, which is of great importance to help health professionals make decisions.

For each sample collected, the *E-Nose* performs a collection protocol based on three categories of actions, aspiration, stabilization, and purge (cleaning step) (as seen in Fig. 3c), where the completion of all three characterizes the completion of a cycle. For each sample, a volume of three readings per second is collected for 20 seconds in the aspiration phase, for 60s in the stabilization stage, and another 60s in the cleaning phase, totaling an average of 420 readings per cycle in each sensor (for each sample, it is a predefined number of cycles is performed). Considering that numerous samples of the same species are needed to obtain diversity in the data (so that the AI models can satisfactorily learn the patterns of each species), a relevant amount of data was collected in this first step, with 20,189 instances of *C. albicans* (3 isolates - ATCC 14053, URM8368, URM8369), 19,068 of *C. glabrata* (1 isolate - URM6393), 6,989 of *C. haemulonii* (1 isolate - URM6555), 7,067of *C. kodamaea ohmeri* (1 isolate - URM6935), 17,255 of *C. krusei* (3 isolates - ATCC 6258, URM8371, URM6391) and 20,234 of *C. parapsilosis* (3 isolates - ATCC 22019, URM7049, URM7048), totaling 90,802 samples collected in approximately 514 cycles with cultures on different days. There are cycles with different sizes due to inconsistent reading in the E-nose. To solve this, it was necessary to match the cycle sizes, explained in more detail in the Sample classification process section ([Anonymous]-URM is a culture collection affiliated with the Word Federation for Culture Collections).

After the construction of the first version of the database, the need to carry out an analysis of the data was identified, seeking to observe the existence of behaviors or indications of patterns for the different sensors related to each of the species. In addition, this initial check was essential to identify strategies for cleaning and restructuring the base to make its use viable by the learning models.

### Data analysis and processing

After generating the data, a descriptive analysis was performed to understand better its behavior and which AI models may be more suitable for identifying the patterns generated by the samples. For this, it was first necessary to analyze and preview the data to get an idea of how they would be about each sensor for each collection of *Candida* spp. After that point, a new database was constructed with the data set of all species collected, with only the sensors considered significant, and with the addition of new columns for labeling the samples about their species and culture time. Another critical point in this information visualization step was using UMAP (Uniform Manifold Approximation and Projection) and PCA (Principal Component Analysis). These two-dimensionality reducers helped to understand the grouping of data better. In this sense, as initial steps for the pre-processing and visualization of information, four relevant points were verified about the data:

- If all sensor data for the same species have similar behavior;
- If there are differences in information between the same species at different collection times;
- If there is a predominance of sensors by species;
- Whether there is a clear division between the data and how it is grouped.

Some graphs with data from all sensors related to the collections of each *Candida* species were generated to analyze the first point. In these, the wave patterns of each collection were observed, following the chronological order of reading, visualized in Fig. 3d for *C. albicans* data.

As can be seen, each of the sensors has a specific wave pattern, varying in well-defined intervals. Some reading peaks in some regions can signal detection errors by the sensors, indicating the presence of possible outliers. Pressure and humidity sensors have an almost constant reading cycle, not interfering at any time with the reading pattern of other gas sensors. The temperature sensor, despite fluctuating a little at some points, also does not interfere with the reading of the other devices, which may be an indication that the alteration of these parameters does not cause, in this case, any interference in the captures of the others sensors, can be removed from the analysis.

Another important point for this initial analysis is identifying differences between data from the same species but for different collection times. This point helps visually determine if there are significant differences between the readings performed with cultures from different days because the earlier the reading patterns are identified, the better the decision-making process. Fig. 3e and f shows data from one-day and two-day readings for the species *C. krusei*.

As can be seen in Figs. 3e and f, there is a slight distinction between the amplitudes of the waves concerning some sensors from one day to the next. This demonstrates that these devices have a difference in resistance of the volatiles between day 1 and day 2. One hypothesis is that the concentration of gases released by this species changes over time, decreasing in some cases and increasing in others, contributing to the differences in patterns between distinct days. Through this analysis, it is possible to focus on the early cycles of culture analysis, streamlining the decision-making process.

The third point is the possibility of a predominance of a particular sensor per species. This can indicate which sensor can differentiate itself more about each *Candida* species, contributing to the distinction of patterns and selection of the features used in the database consumed by the classification models.

Some experiments show that the behavior of sensors is based on the resistance caused by the gases emitted by each species at the time of reading by the Electronic Nose. Seeking to identify a predominance of a sensor over the species, it was noted that the TGS2602 and TGS822 sensors have a greater amount of readings spread over different resistance (Ohm) levels for *C. parapsilosis*, with the values of the other *Candida* in regions very similar but quite different from *C. parapsilosis*. The opposite occurred with the TGS2611 and TGS823 sensors, where the other samples had more distributed resistances and *C. parapsilosis* more focused on a region. This all shows that some sensors have predominance about some species; however, to identify different levels of resistance about the other, all reading values end up being relevant, as together they become important characteristics for identifying patterns by models.

**Figure 4:**
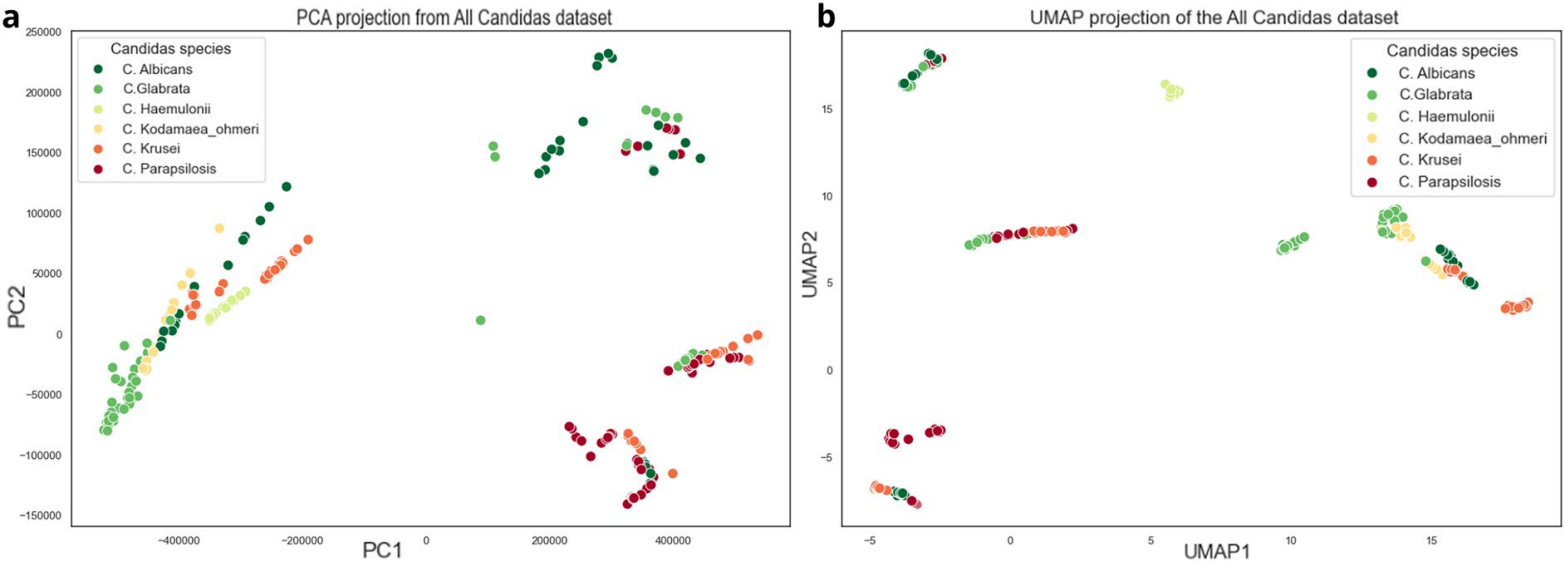
Two dimensions from Principal Component Analysis (**a**) and Uniform Manifold Approximation and Projection for Dimension Reduction (**b**).

After analyzing the data for each species and sensor separately, the need to understand how the entire dataset was grouped was identified. For this, two techniques for dimensionality reduction were applied: PCA (Principal Component Analysis) and UMAP (Uniform Manifold Approximation and Projection). In the case of PCA, according to^42^, its main objective is to extract relevant information from a set of tabulated data and convert it into a new set of orthogonal variables called Principal Components. In this sense, it is possible to display similarity patterns in the instances and variables as components in a graphical map. On the other hand, the UMAP, according to^43^, is an innovative technique of dimensionality reduction that is based on a theoretical structure of Riemannian geometry and algebraic topology, which makes the results derived from its reduction scalable and easily used on accurate data. Unlike PCA, it performs dimension reduction non-linearly, trying to keep similar cases close together and different cases separate. This study applied a two-dimensional decrease for both techniques, which can be analyzed in Fig. 4.

Analyzing the two projections, we can see small groups built by each species. In the case of PCA, the standard difference from *C. parapsilosis*, *C. albicans* and *C. glabara* for the other *Candida* is evident, as their points are well dispersed from the additional data group, with some samples separated from the leading group. This demonstrates that this species has very particular characteristics and can probably be distinguished by IA models. Although the other species are concentrated in a single region, they are well separated, with not much visible shuffling between them. One visual problem is the existence of the same group in different parts of the PCA image. Some models can find issues to distinguish this behavior. In the graph generated by UMAP, it is already possible to see a separation of the data, with groups of species being made in different regions of the graph. This is explained by how UMAP deals with reduction through algebraic topology and similarity measures. It is essential to highlight that, despite not being grouped in the same region of the graph, species with similar characteristics end up staying close to each other and, because they have very different reading averages within the same species - due to the differences in sensor readings - the same species may contain data that are not very close, considering that this method does not seek its resizing based on the main components, but on similarity measures.

Finally, knowing how the data are arranged and grouped, the base was prepared for use by Time Series models, modified to 2 dimensions, one of the few ones withstand by most models in this segment. From there, experiments with the models were started, which will be detailed in the following sections.

### Sample classification process

With clean and structured data, the models were selected based on the results of the data visualization phase and the study on *Inception Time*^34^, which compares it with other state-of-the-art models, including its predecessors, the Hierarchical Vote Collective of Transformation-based Ensembles 1^44^ and 2^45^ (HIVE-COTE 1 and HIVE-COTE 2). The information visualization showed that the data do not overlap and have a single division between them, so there are not many restrictions on which categories of models to use. Thus, in addition to the techniques already mentioned, the K-Neighbors Time Series Classifier (KNN) was also introduced in the experiments, which implements the K-nearest neighbors for time series^46^, the Time Series Forest Classifier (TSFC), implementation of a Time Series Forest using intervals^36^, the Shapelet Transform Classifier (STC), which uses transformed discriminatory subseries as a classifier^47^, the Random Interval Spectral Ensemble (RISE), built based on trees and different sets of partial and automatic correlation of features^37^, the ROCKET Classifier (ROCKET)^35^ and BOSS Ensemble (BOSS)^48^, all Time Series models that will be used as a classifier, due to the temporal characteristic of the data, translated through the parameter *culture_day* from the base.

As previously mentioned, a total of 90,802 readings of the six species of *Candida* were collected in about 514 cycles; however, to obtain a “smell impression” from data, it was necessary to concatenate all readings of all sensors of a cycle in one row of the dataset, resulting in a new set of 397 instances with 821 columns (now, each sample is related to a cycle). Therefore, the base was divided into training, validation, and test sets, with 60% for the first (238 cycles) and 20% for the other (79 and 80 cycles).

Stratified cross-validation is used to maintain a homogenized proportion of data sampling to ensure that the training set can represent the entire population, avoiding sample bias^33^. For each subset used in training, results were obtained for five metrics: accuracy, recall (sensitivity), F1-Score, precision, and specificity^49^. Accuracy measures the proportion of correct model predictions over the evaluated examples. Recall (sensitivity) is applied to measure the portion of patterns correctly identified by the classification model. Specificity is used to test the ability to determine healthy cases accurately. On the other hand, precision is applied to measure the quantity of correctly predicted positive patterns based on the total amount of predicted patterns in a positive class. Finally, the F1-Score or F1-measure portrays the harmonic mean between precision and recall values^38^. All these metrics are calculated based on the values of true positive (TP), false positive (FP), false negative (FN), and true negative (TN), obtained after the crossing of predicted values with the actual values of each class.

Therefore, at the end of the experimentation process, a statistical analysis using the Shapiro-Wilk normality test, the Kruskal-Wallis non-parametric test, and the Nemenyi post-hoc test was applied to understand the statistical significance between the means of the results and highlight the difference between the models tested, which are detailed in the Results and discussions section.

## Data Availability

**Accession codes and database**: The code and datasets generated and analyzed during the current study are available in the ’CandidaIdentification’ repository: https://github.com/michaellopes16/CandidaIdentification.git. The research described in the article does not use human tissue, only ATCC standard samples.
